# Microfluidic chip-based technologies: emerging platforms for cancer diagnosis

**DOI:** 10.1186/1472-6750-13-76

**Published:** 2013-09-27

**Authors:** Li Ying, Qi Wang

**Affiliations:** 1 Department of Gastroenterology Medicine, The Second Hospital Affiliated to Dalian Medical University, Dalian, China; 2 Department of Respiratory Medicine, The Second Hospital Affiliated to Dalian Medical University, Dalian, China

**Keywords:** Microfluidics, Diagnosis, Oncology, Gene, Cancer biomarker

## Abstract

The development of early and personalized diagnostic protocols is considered the most promising avenue to decrease mortality from cancer and improve outcome. The emerging microfluidic-based analyzing platforms hold high promises to fulfill high-throughput and high-precision screening with reduced equipment cost and low analysis time, as compared to traditional bulky counterparts in bench-top laboratories. This article overviewed the potential applications of microfluidic technologies for detection and monitoring of cancer through nucleic acid and protein biomarker analysis. The implications of the technologies in cancer cytology that can provide functional personalized diagnosis were highlighted. Finally, the future niches for using microfluidic-based systems in tumor screening were briefly discussed.

## Introduction

Although much progress has been made in the diagnosis and treatment of malignancy, cancer is still the most common cause of death worldwide. Most patients with cancer have symptoms and distant metastases when diagnosed, which makes it more difficult to successfully treat the disease. Therefore, accurate prediction and diagnosis at early time are the most critical issues in cancers. Recently, significant efforts have been put into finding informative cancer biomarkers that can contribute to the establishment of cancer diagnosis [1]. The biomarkers encompass mutated DNAs and RNAs, secreted proteins, and tumor cells (e.g., circulating tumor cells, tumor stem cells) [1]. Ideally, the measurements of cancer biomarker screening are supposed to be done at high accuracy with automation and cheaply at point-of-care to reduce costs. Unfortunately, the methods performed typically in traditional bench-top laboratories are usually not amenable to high-throughput screening which are responsible for large-scale “-omics” studies and have been approaching a plateau in almost all fields of oncology. In the past decades, vigorous efforts have been undertaken to develop new and robust laboratory tests. Gained from the advances of micro- and nano-fabrication approaches, microfluidic technologies, also referred to as Lab-on-a-Chip (LOC) or micro-total analysis systems (μTAS), offer tremendous hope in both point-of-care cancer biomarker measurements and personalized diagnostic strategies and have evolved state-of-the-art devices for cancer research [2,3].

Typically, microfluidic technologies enable the actuation of fluids and manipulation of bioparticles (e.g. DNA, RNA, proteins, and cells) at the microscale [4]. Fluid flow in ultralow dimensions of micrometers is laminar and can be precisely controlled by adjusting the flow rate. The distinct property gives rise to more efficient and accurate mass delivery to cancer cells in controlled time and space [5,6]. Also, because microfluidic platforms have scalable sizes with most biological macromolecules, cells and blood vessels, they provide unique functionality for the design and remodeling of precise scaffolds, which mimick the physiological tumor microenvironment [7]. The characteristics are especially suitable for cancer cytology research considering that cancer cells have been found to present enhanced viability, invasion, and variable therapeutic response when embedded in microfluidic culture systems [8,9]. Most importantly, the microfluidic-based studies pave the way for integrated genomic, proteomic, and cytomic analyses to identify hundreds of novel candidate biomarkers potentially involved in tumorigenesis [10,11]. As discussed in this review, microfluidic chips are gaining exclusively interest in the emerging field of systems oncology. The tequniques provide new avenues to facilitate the thorough understanding of the molecular mechanisms of malignancy at the systems-level.

This article presented a current overview about the potential application and perspectives of microfluidic technology in clinical oncology. Notably, due to the wide range and rapid proliferation of the subject matter, the paper was not intended to provide a comprehensive review but to provide the reader with a practical appreciation on the use of microfluidic devices in clinical cancer research.

## Review

### Microfluidic-based gene analysis

Genetic changes in cancer cells which lead to altered gene expression patterns can be utilized as biomarkers for detection and diagnosis of cancer [12]. Gene expression profiles have shown dramatic correlations with tumor development, progression, intra- and extra-vascular invasiveness, and patient outcome when applied to biopsy samples. However, the profiles of somatic mutations in different tumors are much more complicated than we can imagine and the complexity of mutational heterogeneity makes it difficult to identify key cancer genes that could provide sufficient diagnostic utility [12,13]. Recently, microfluidic chip based devices have been increasingly heralded as robust techniques for genomic diagnosis, holding promises to profile tumor genomic sequences and interpret the implication of such sequences in e.g. cancer origin, or progression.

**Figure 1:**
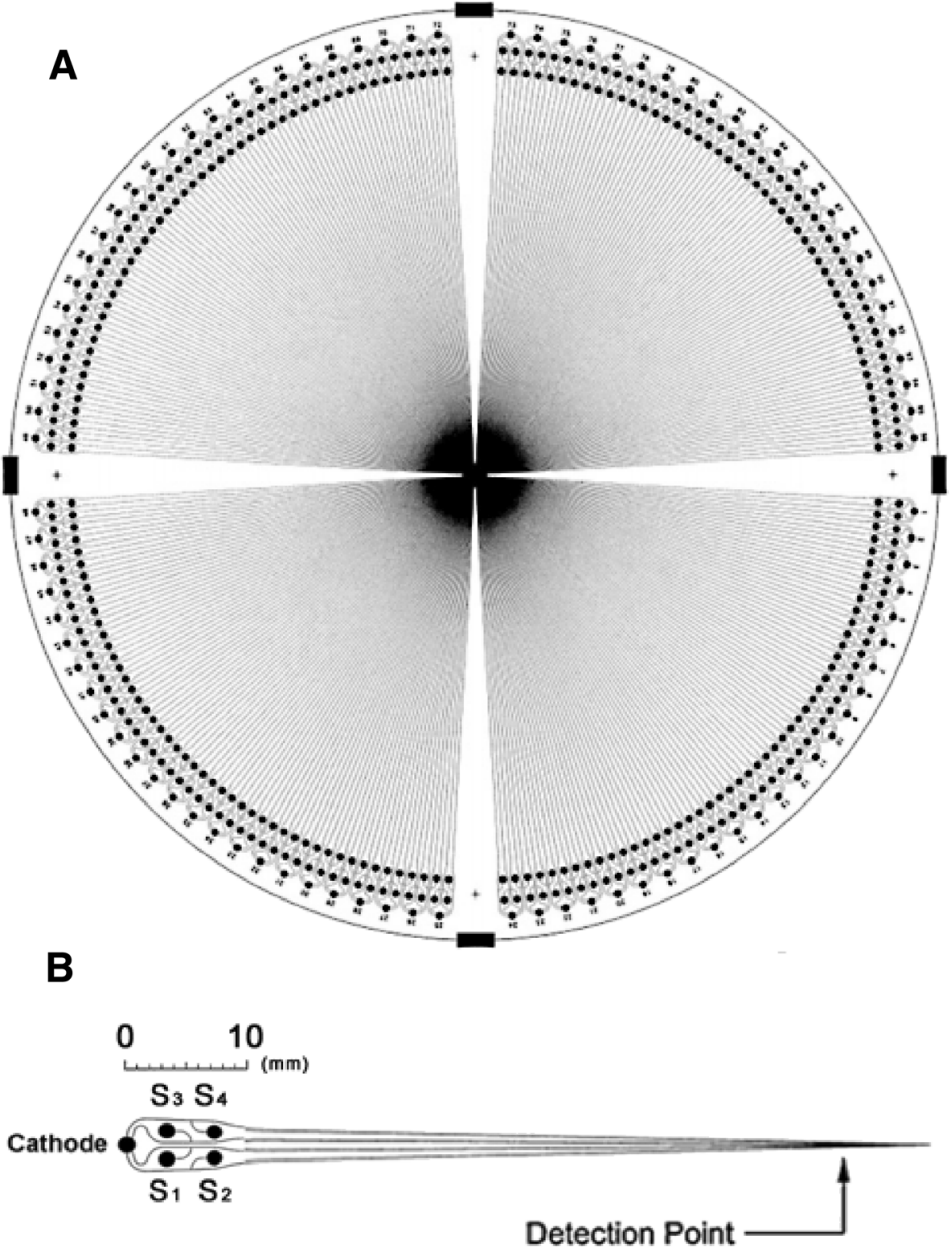
**High-throughput μCAE device. (A)** Layout of the 384-channel μCAE device. **(B)** Expanded view of a single quartet of channels and their injectors. The channels of a quartet share a common cathode reservoir. The injector was fabricated with individual sample reservoirs (S1–S4). (Reproduced with permission from Ref [17]).

The development of a high-speed, high-throughput DNA sequencing method represented the most attractive challenge for clinical molecular diagnostics, especially mutation detection and genetic screening. Capillary array electrophoresis (CAE) appears to be a prime candidate to be employed among various DNA sequencing methods [14]. Since considerable advances in microtechnology allow smaller and denser microchannel arrays with complex turn geometries to be fabricated, microfluidic-based CAE (μCAE) chips for multiplex sequencing of oncogenes have been at the center of scientific interest. For example, a microfluidic gene array was used to indicate the down-regulated gene expression profiling of mTOR-associated tumor suppressor genes in ovarian cancer patients [15]. A spatial temperature gradient μCAE system was presented for quantitative detection of low-abundance mutation DNA [16]. The system obtained accurate separation and detection of K-ras gene from paraffin tissue sections of colorectal cancer. Indeed, rectilinear and radial channels for parallel high-throughput analysis have been designed on microfluidic devices in DNA assays (Figure 1). Integrating microfluidic channels on chips provide a potential for reducing DNA diffusion time and optimizing hybridization conditions, such as temperature, ionic strength, and denaturant concentration.

The use of microchips as a miniaturized polymerase chain reaction (PCR) platform realizes an autonomous lab-on-a-chip system, which can reduce the overall analysis cost as well as preventing the potential for sample cross contamination, and importantly, provide the more accurate and high-throughput means of cancer gene research [18]. Generally, two kind of configurations are used in PCR microfluidic devices: (1) a stationary configuration as in conventional PCR wherein the sample is held in a microchamber and the temperature of the chamber is cycled; (2) a flow-through configuration wherein the sample flows through different thermal zones, which are responsible for distinctive processes, such as denaturation, annealing and extension [2]. A continuous-flow PCR microfluidic chip was recently developed by employing a water cooling thermocycling within the chip center whereby the sequence of denaturation, annealing, and extension was created due to the forced convection effect and DNA fragments with different lengths (372 bp and 478 bp) were successfully amplified [19]. More efforts also include integrating PCR with sample preparation steps such as DNA purification. A microfluidic sequencing enrichment method was reported for oncogene identification on a genome-scale level [10]. In this study, a 480 Kb exome subset of 115 cancer-related genes were captured. The device also revealed an analysis of reference single nucleotide polymorphisms (SNP) with a sensitivity of up to 93 % and a specificity of 98.2% or higher. Due to advantages of efficient sample handling and in vitro transcription reactions, on-chip RT-PCR is also successfully applied in cancer research. For example, a multiplex RT-PCR (mRT-PCR) assay was developed for rapid screening of alterations in prostate cancer genes at miRNA levels. This design was characterized by its integration with microfluidic-based on-chip electrophoresis [20]. Multiplexing made the approach superior to gene-by-gene analysis by quantitative RT-PCR and more reliable for analyzing rare amounts of RNA from clinical tumor biopsies. Similarly, a real-time quantitative PCR (qPCR)-based microfluidic platform was used to evaluate the miRNA expression levels in lung cancer cell lines [21]. This approach exhibited a throughput that was 5 to 20 times higher and a sample and reagent usage that was approximately 50-100 times lower than conventional assays.

### Microfluidic-based protein analysis

Although gene analysis has opened up a few applications in cancer research, discrepant changes in gene expression might not be reflected accurately to the level of protein expression or practical function [22]. Protein cancer biomarker is directly derived from the oncogene and it in turn leverages both the gene expression and cellular metabolism. For a better understanding of cancers and their refined classification at the systems-level, the analysis of protein expression and function is of particular importance. However, the identification of proteins expressed in a given sample has presented unique analytical challenges, including significant molecular diversity, dynamic range of proteins at the cellular level, extreme complexity of post-translational modifications and a tendency to adsorb to solid surfaces [23]. Herein, we focused on microfluidic screening of biomarker proteins that can be used not only for indicating the onset, progression and invasion of cancer but for directing personalized cancer diagnosis and treatment monitoring.

**Figure 2:**
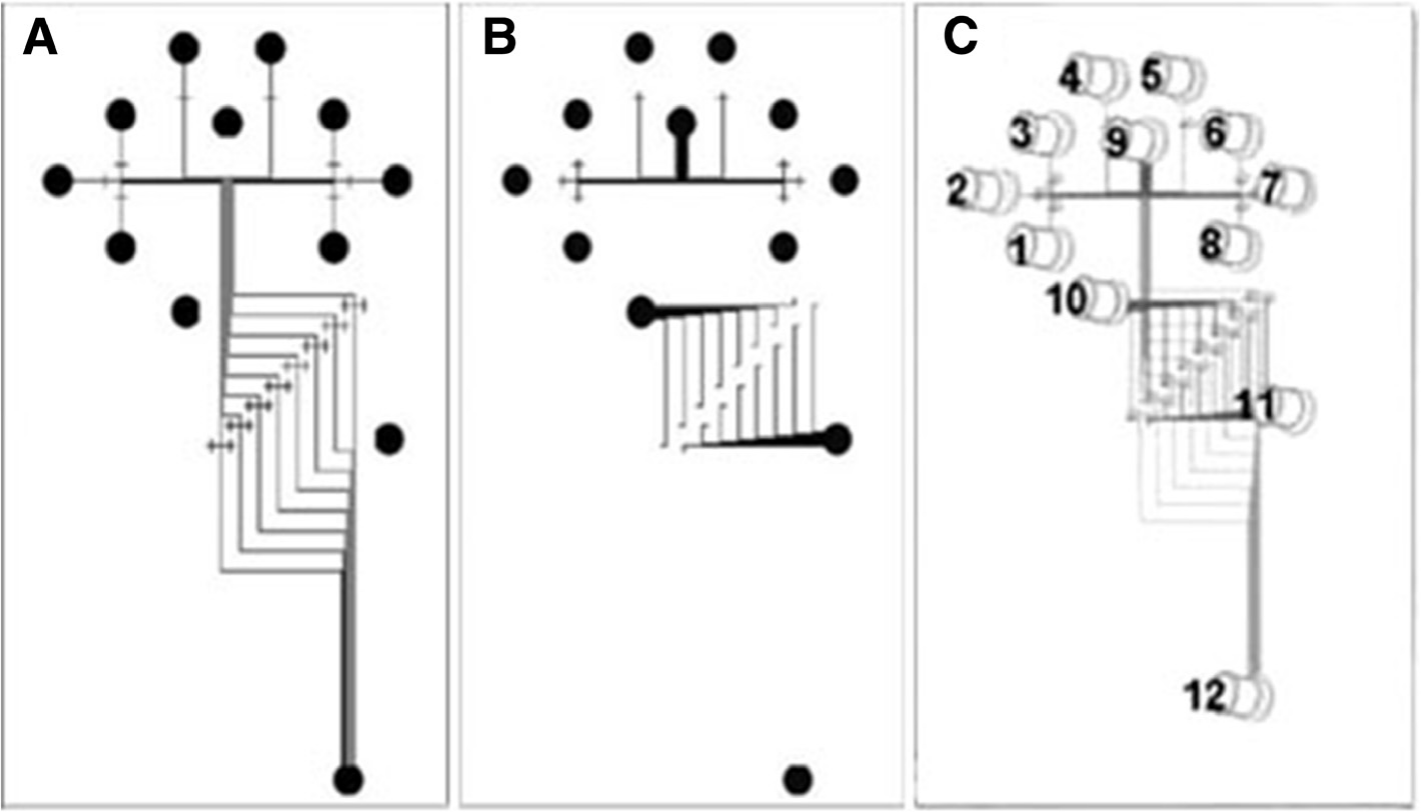
**Schematic of the integrated microchip CAE device. (A)** the bottom layer; **(B)** the top layer; **(C)** a complete device. Reservoirs 1-8 are sample inlets and reservoir 9 is the fluorescent label inlet. All parallel electrophoresis units share the same electrophoresis reservoirs (reservoirs 10-12: buffer inlet, buffer waste, and separation waste). (Reproduced with permission from Ref [24]).

Integrated CAE microchips have potential to address needs in high-throughput assays of proteins, in particular, capturing and quantifying cancer biomarkers. A polymethyl methacrylate (PMMA) microchip CAE system was fabricated with integrated on-chip fluorescence derivatization function [24]. The device consisted of 8 parallel lanes, allowing up to 8 different samples to be labeled and separated simultaneously. The system was demonstrated to be capable of separating a cancer-related protein from the mixtures of model biomarkers in parallel (Figure 2). Recently, a microfluidic chip was reported to realize miniaturized Western blotting for protein assay. The device was interfaced to a moving membrane so that protein complexes were separated by sieving electrophoresis in discrete zones as they migrate from the chip. Capture of proteins were completed in 2 min with 4 × 10^4^ theoretical plates at 460 V/cm. Total analysis time including immunoassay was nearly half an hour for a single sample. Further improvements in throughput could be aquired by increasing more sample reservoirs or parallel channels on the chip [25].

For various solid malignancies, the routine diagnostic methods rely on qualitative tissue immunohistochemistry (IHC). IHC allows the identification of proteins of interest and provides information on protein localization and tissue morphology. However, it is now apparent that panels of protein cancer biomarkers, as opposed to single biomarkers, will be necessary for reliable cancer detection and monitoring [26]. Unfortunately, multiparametric analyses via conventional IHC are technologically challenging and rarely used clinically. Although co-expression for biomarkers was realized by direct and indirect sequential staining methods with molecular dyes and quantum dots, multicolor IHC studies suffered several drawbacks, including low stability of primary antibodies, difficult conjugation of probe to antibodies, high cost of reagents, and cross-over nonspecific binding of secondary probes [27]. Microfluidic-based IHC platforms have been proposed as robust quantitative tools to further enhance throughput of cancer biomarker multiplexing [28,29]. A microfluidic multiplexed IHC device was developed via interfacing specimens slide with different reaction microchannels on a chip [29]. Through this platform, several cancer-related proteins could be simultaneously investigated on a single tumor tissue section, which facilitated histopathological diagnosis and individualized therapy of cancer.

Over the past century, mass spectrometry (MS) has evolved into a primary analytical tool in the proteomics area because of its sensitivity and accuracy. Advances in MS have been achieved by combining with several techniques, including gas and liquid chromatography, CE, electrospray ionization (ESI) and matrix-assisted laser desorption ionization (MALDI) [30,31]. The coupling of microfluidics to multi-techniques of MS analysis has shown more immediate promise to achieve signatures for cancer research by addressing issues like speed, throughput, technically complex and cost efficiency. For instance, a chip-based, reversed-phase liquid chromatography-MALDI-MS analysis platform was utilized for quantification of glycomic-profile changes as cancer-biomarker discovery [32]. Another mode integrating chip-CE to nano-ESI MS realized higher sensitivity of the trace-level protein biomarker detection. [33] Disposable nanospray emitters and the chip itself as the MALDI target made the interface more suitable for point-of-care [33]. Besides, the innovative chip devices produced an extremely low dead volume connection which normally has a significant impact on analytic results by classic MS. An off-line coupling microchips to MALDI, called integrated selective enrichment target technology, was developed to provide faster multiplex analysis of proteomic samples with high-sensitivity by minimizing the number of sample transfers and the total surface area [34].

### Cancer cytology analysis with microfluidic systems

The research of cancer cytology is the most straightforward way for screening and monitoring precancer and cancer lesions [35]. Bioanalysis at cellular level gives direct information of a single cancer cell or cell-to-cell communication within a complex tumor microenvironment. Due to their compatible sizes with cells, microfluidic systems are ideal platforms for cell manipulation and analysis in vitro.

#### *Cell sorting*

The accurate separation of tumor cell subpopulations is a preliminary but often tedious step for cancer cell biology. Currently, the most widely used methods of cell sorting are fluorescence-activated cell sorting (FACS) and magnetically-activated cell sorting (MACS) [36-38]. The technologies provide several advantages, such as rapid single-cell level separation, enrichment and purification of rare cells (e.g. tumor stem cells and circulating tumor cells), and high-speed multiparameter analysis [37,39,40]. Traditional cell sorting systems, however, are still cumbersome and costly, which limit the full utilization of these systems in various biomedical research and clinical applications [41]. In this regard, chip-based separation formats are especially powerful. In recent years, numerous microfluidic cell sorters have been designed, which are based on various sorting principles, including electric, magnetic, hydrodynamic and optical mechanisms [41-44].

**Figure 3:**
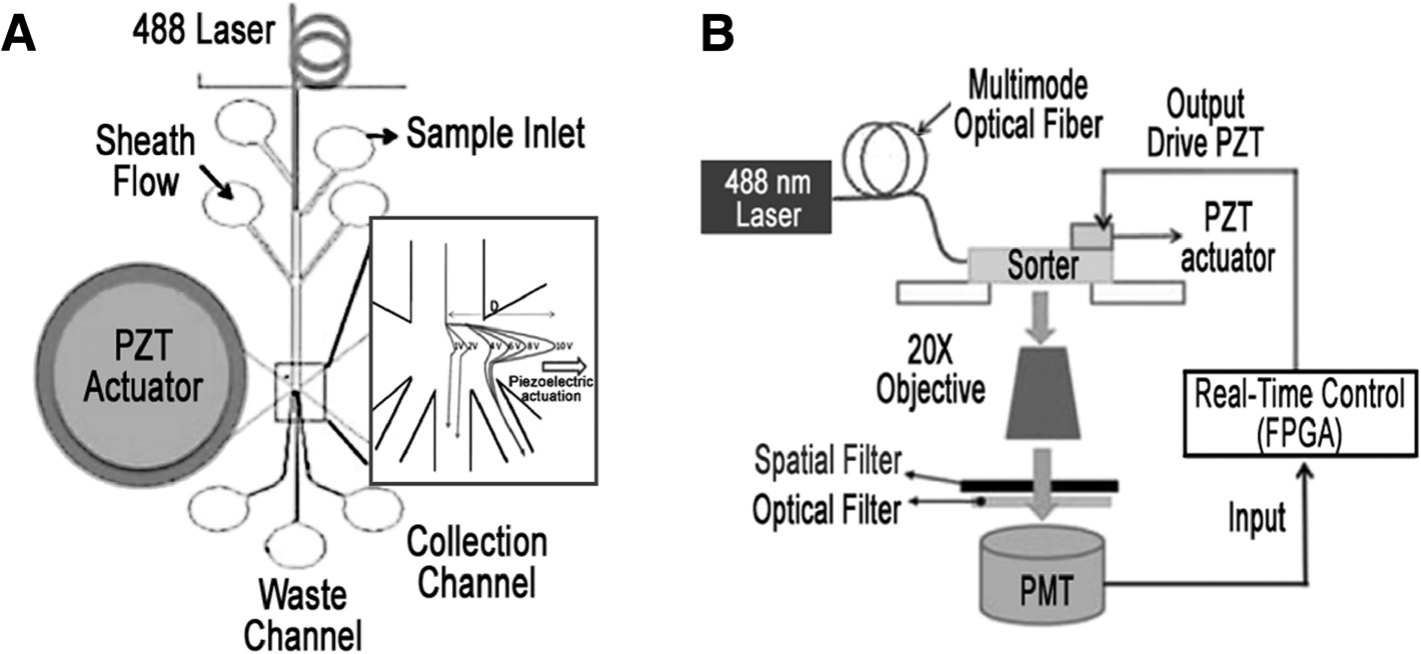
**A fully integrated μFACS system coupling various sorting principles. (A)** Structure of the μFACS device with microfluidic channels and a sorting PZT actuator. The cell trajectory plot under different voltage magnitudes to the PZT actuator was observed with superimposing photos taken by a high-speed CMOS camera. **(B)** Schematic of the overall μFACS system. The laser-induced fluorescence from the stained cells was collected by a microscope objective lens. Fluorescence signals were modulated into a time-domain signal of similar waveform via a spatial filters at the image plane and were registered by the photomultiplier tube (PMT). The output signal from the PMT was imported into the electronic control system for real-time processing. (Reproduced with permission from Ref [41]).

Significant progress has so far been made toward developing microfabricated FACS (μFACS), enabling high screening throughput and high purity enrichment. Optical force was firstly developed in a μFACS system to sort fluorescent Hela cells [45]. The system allowed a rapid, high efficient control of cell isolation while no significant influence on cell viability, activation state, and functionality occurred. Recently, a microfluidic cell sorter was used for circulating tumor cell detection, on-chip culture and subsequent analyses through cell retrieval [46]. Also, a hydrodynamic driving μFACS was further reported with a hydrodynamic gating valve permitting automatic on-chip sorting with an average purity of 93%, a recovery rate of 93%, and a viability of 94% [47]. Notably, a fully integrated μFACS system could realize coupling optics, acoustics and electronics system on a single microfluidic platform (Figure 3) [41]. The device has a main microfluidic channel followed by a sorting junction and three sub-channels for collecting waste and samples. The laser light was delivered to the device by the optical fiber and guided by optofluidic waveguide. A piezoelectric lead-zirconate-titanate (PZT) actuator was integrated in the sorting junction. As the PZT actuator bent down, the cell of interest flew to the sorting channel while the non-targeted cell flew to the waste channel. By using the innovative actuation mechanisms, single cell manipulation was achieved at a high throughput with high purification enrichment factor. In addition, the requirement of a low voltage (<10 V) and the low consumption of power made it much more suitable for point-of-care diagnostic applications.

Alternatively, many other types of microfluidic-based sorting devices have also been proposed lately. Microsystem based MACS technologies (μMACS) allows high-throughput sorting of target cancer cells from the clinical samples based on target-specific affinity markers [42,48]. Lien *et al* reported a three-dimensional (3D) microfluidic platform for rapid isolation and detection of cancer cells from a large sample volume [42]. With the incorporation of surface-modified magnetic beads, target cancer cells were specifically recognized and were subsequently isolated and purified with a high sensitivity. This system presented a promising platform for the analysis of tumor genetic marker utilizing the built-in nucleic acid amplification module. A microfluidic dielectrophoresis (DEP)-activated cell sorter was developed for the isolation of human leukemia cells from dilute blood samples [49]. DEP has advantages of low-voltage operation and selectivity for driving cells by frequency. A microfluidic fluorescence-activated droplet sorter (μFADS) was also reported [50]. In this study, single cells were encapsulated in emulsion droplets and were subsequently sorted using DEP. This sorting approach was expected to provide a new concept for studies of rare phenotypes within heterogeneous populations of cells. A microfluidic surface acoustic wave actuated cell sorter was patterned to isolate melanoma cells [51]. The device represented the combined advantages of both the FACS and FADS. In addition, it saved the trouble of prior encapsulation into liquid droplet compartments. Most importantly, the low shear forces of this device ensured that no significant damage occurred to the cells during the sorting procedures, which allowed subsequent applications for other bioassays. Recently, a microfluidic acoustophoretic chip was also used to sort cancer cells based on cell mechanical properties and cell size [52]. The chip could produce acoustic-standing-waves under which cancer cells with different mechanical properties show different transmission intensity. The acoustophoresis chip had a straight flow channel with a piezoelectric transducer attached at the bottom. By using the model, the trajectories of cells in the channel under acoustic standing wave excitation which showed different metastatic capacities were calculated.

#### *Single cell analysis*

The heterogeneity of cancer cells which can dramatically influence cell fate and biological responses has led to increased interest in individual cell studies. A number of single cell analysis approaches have been established, among which flow cytometry and fluorescence microscopy are most commonly employed [53]. These methods, however, have challenges with complexity, time and sample consumption, and most importantly, inefficiencies in manipulating single cells in real time [53]. For the purpose of achieving total single cell analysis (that is, analysis of whole cells or cell lysates or both), the development of high-throughput systems capable of handling and analyzing individual cells is essential. In the past decade years, microfluidics has been rapidly developed into a powerful approach capable of integrating multiple functions for single cell analysis [54].

Intact single-cell analysis Cancer cell populations represent intrinsically variable and stochastic systems characterized by high levels of spatiotemporal complexity [55]. However, conventional cell-based assays can only measure the average response from a population of cells and provide statistically meaningful data. This simplification can lead to a misleading readout. One approach to solve this dilemma is to analyze the whole population at individual cell level.

**Figure 4:**
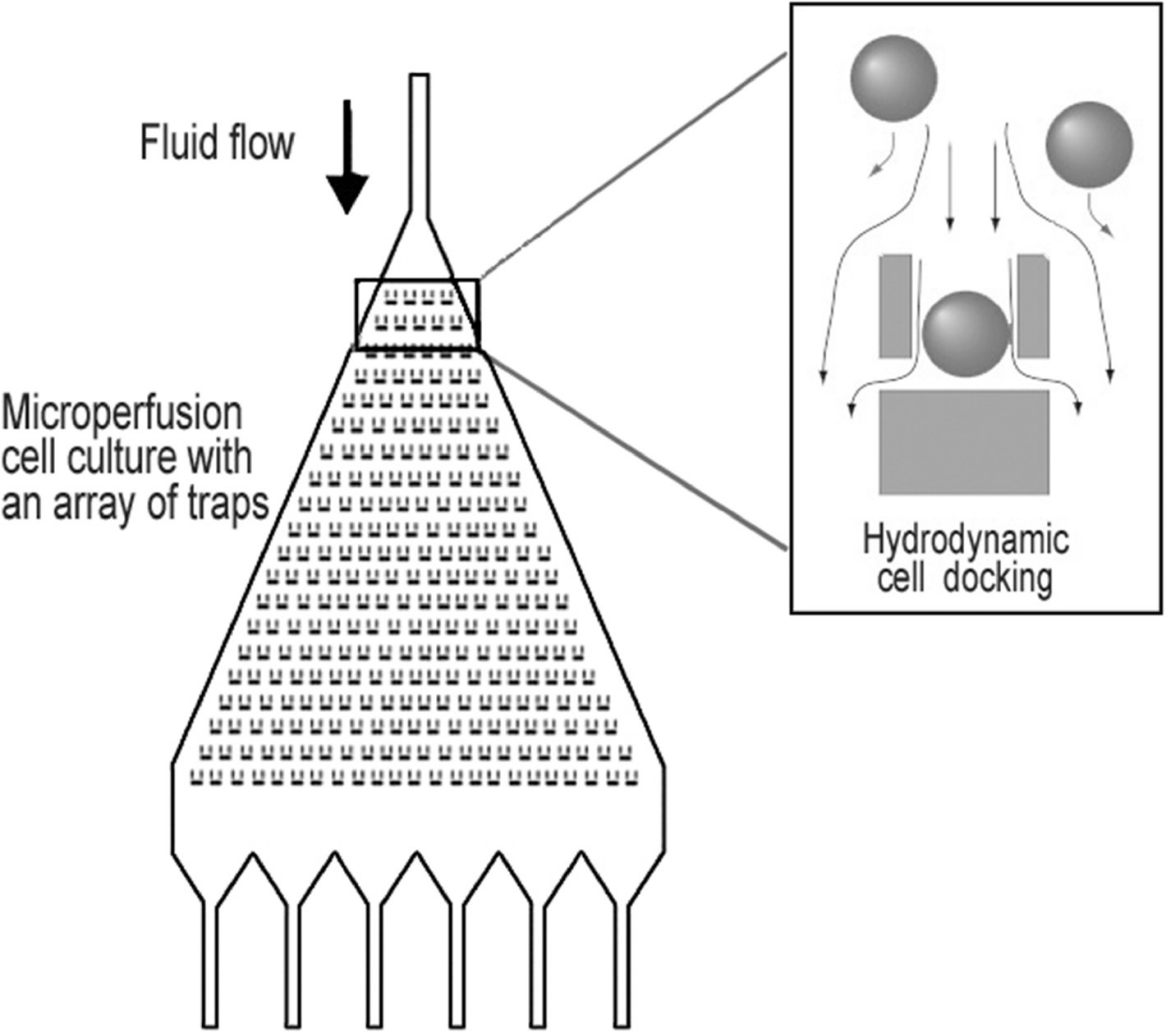
**Design of the microfluidic chip with a triangular chamber for a hydrodynamic single-cell docking and microperfusion culture.** A low-density cell positioning array was fabricated in a biocompatible elastomer, polydimethylsiloxane (PDMS), and bonded to a glass substrate. The microfluidic array cytometer allowed for a gentle trapping of single live cells for prolonged periods of time. (Reproduced with permission from Ref [58]).

Rapid analysis and characterization of primary cancer cells using microfluidic technology has aroused many interest for clinical research of oncology. At present, microfluidic flow cytometry (μFCM) has offered promising avenues that leverages the multiparameter and high-speed measurements owing to their established nature. For instance, McKenna *et al* reported a multi-channel parallel μFCM that was based on analog detection combined with parallel microfluidics [56]. The multiple microfluidic flow channels allowed the independent optimization of cell count rate, samples per minute, and signal-to-noise ratio, proving the feasibility in high content screening. Nevertheless, μFCM still suffers from a lack of capabilities for dynamic analysis of single living cells [53,57]. This leads to the development of new LOC designs, that is, living cell microfluidic arrays. These unique formats allow creating positioned arrays to arrange the cells in a spatially defined pattern and as such are ideal platforms for kinetic and multivariate analysis on a single cell level, which is particularly useful for understanding cell-to-cell variability as well as cancer cell decision making [57]. Furthermore, unlike static cell microarrays, microfluidic cell arrays that allow for fabrication of parallelized and fully addressable arrays facilitate physiologically relevant exchange of stimulants and metabolites and thus attain a precise spatiotemporal control of cell behavior over the artificial on-chip microenvironment (Figure 4) [58].

Alternatively, an innovative design was developed using a miniaturized diagnostic magnetic resonance (DMR) system for multiplexed, quantitative and rapid analysis of cancer cells [59]. With the chip-based biosensor, cancer cells were detected by targeting cell surface markers. The resulting DMR systems were anticipated to be a truly portable, easy-to-use and low-cost device for point-of-care use. Subsequently, a miniaturized nuclear magnetic resonance probe was used for detection and molecular profiling of cancer cells [60]. The distinct advantage of the method was its potential for detecting rare cancer cells in the unprocessed cancer samples in a few minutes. Notably, miniature label-free cell analysis systems have been used to interrogate whole cells. Typical examples of label-free on chip cytometry were spectral impedance measurements which provided information on cell size, membrane capacitance, and cytoplasm conductivity as a function of frequency. The amplitude, opacity, and phase information can be used to discriminate different cell populations without the use of cell markers [61]. Recently, a label-free optofluidic intracavity spectroscopy was developed which was integrated with a microfluidic optical resonator to distinguish hemangiosarcoma cells from peripheral blood monocytes [62]. The results revealed the differentiation of HAS cells with 95% sensitivity and 98% specificity.

Cell lysate analysis Cell Lysis is a crucial step upon which the subsequent analysis of extracted intracellular components depends. Mechanical cell lysis system was fabricated in early stage by using a microfluidic filter with nano-barbs [63]. Despite the efficiency of lysing the cells, this method failed to provide sufficient proteins for further analysis because of the resultant cell debris. For overcoming the defects, magnetic field and a centrifugation system which was used for sample homogenization were further employed [64]. In addition, chemical lysis method was developed based on the use of enzymes (lysozymes) or detergent solutions and had the advantage of easy-integration by dispensing the chemical from reservoirs [65]. However, the lysis condition that interfered with downstream molecular biology should be considered. Still, microfluidic electrical lysis process was developed for the analysis of subcellular materials of human carcinoma cells [66,67]. This lysis method was characterized by the selective performance towards plasma membrane with fast speed and simple operation while leaving organelle membrane undamaged. Laser-induced cell lysis techniques have begun to attract much notice which simplify the incorporation of the lysis protocol into a microfluidic chip. The lysis devices involved a pulsed laser beam emitted by an optic instrument to generate shock waves and cavitation bubbles which eventually caused cell rupture.

Stochasticity and complexity in gene expression, protein or metabolite levels contribute to cell-cell variations. Ideally, the relationship between the cellular heterogeneity and cell behavior, which is key to understanding the development, progression, and treatment of cancers should be elucidated at the single-cell level [68]. Substantial developments have been made currently to integrate different protocols on microfluidic platform for single cell analysis. In the analysis of DNA patterns in single cells, a droplet-based microfluidic platform coupled DNA purification with sequencing of multiple gene targets was used [69]. The system enabled detailed studies of mutation co-occurrence and synergy during carcinogenesis. Similarly, a PCR-slide microfluidic system was presented with the restriction enzyme-based single-cell methylation assay [70]. A microfluidic reverse transcription-PCR chip was also utilized to isolate mRNA templates by using beads coated with poly-T nucleotides, as well as to synthesize cDNA from individual cells [71]. More recently, a microfluidic RT-qPCR device was designed capable of performing high-precision measurements of gene transcription as well as single nucleotide variant detection in single cancer cells [72]. The device incorporated all steps of single-cell processing, including cell capture, cell lysis, reverse transcription, and quantitative PCR. For quantitative measurement of single-cell proteins, a microfluidic image cytometry was reported for proteomic analysis of multiple oncogenic signaling proteins of clinical brain tumor specimens [73]. The striking intertumoral and intratumoral heterogeneity characteristic were revealed which further confirmed stochasticity in protein levels can contribute to cell-cell variations. Notably, a microfluidic system integrated on-chip CE was presented for both protein analysis and whole genome amplification (WGA) [74]. This technology provided new clues for studying protein profiles of samples and simultaneously accessing genomic information based on WGA.

## Conclusions and future trends

Microfluidic devices provide numerous advantages over the traditional macroscale methods and slowly emerge as new platforms in a wide range of researches in clinical oncology. At molecular level, the analysis methods of gene biomarkers including gene mutations, cancer-related methylation, and other alterations were firstly introduced which included microfluidic chip based CAE and PCR. Protein biomarker analysis were then reviewed which mainly focused on the interface of microfluidics with IHC, ELISA, and MS. At cellular level, different microfluidic based methods were extensively employed which include cell sorting, cell culture and unique manipulation on isolated single cancer cells. These were, thus, particularly attractive for the clinical and diagnostic laboratories, allowing rapid analysis of only small amounts of patient derived cells. Moreover, the reconstruction of cancer microenvironment on chip provided new vistas for integrated cancer cytomics in clinical diagnostics and drug screening routines.

Regardless of the advancement in the field of micofluidic system, this system remains in its infancy and still has several disadvantages, such as (1) the complicated fabrication processes, for example, the use of valves and pumps to fabricate large arrays of microchannels often require experimental setups and equipment not readily available in most labs; (2) the suitable interfaces for fluid transfer into, within, and out of the microfluidic chip may not be entirely satisfactory especially for portable and field-use systems; (3) the commonly used PMDS for fabricating the microfluidic device present with hydrophobicity and propensity for protein absorption, which may disturb the bioassay results; (4) electrokinetics methods driving fluid flow in microchannels suffer from the limitations of buffers incompatibility, electrolytic bubbles formation, evaporation of solvent and electrophoretic demixing due to different electrophoretic mobility; (5) the use of microfluidic systems often requires expertise which is not the part of training for the life science professionals.

Several of the issues described above has raised a recurring theme throughout extensive applications of microfluidics that device integration for true on-chip functionality without requiring cumbersome and costly external ancillary equipment such as power supplies, capillary pumps, lasers, and mass spectrometers can be practically used and more widely adopted in a variety of laboratory, or commercial settings. These tests afforded by microfluidic-based lab-on-a-chip devices can be administered at a patient’s locale and even by the patient himself, offering not only convenience but significantly more rapid diagnosis than conventional lab-based testing. In the long term, a detection system can be envisioned that consists of an inexpensive, single-use, disposable cassette in conjunction with a portable analyzer. Specially for cancer detection, the microfluidic system will provide a means for cancer typing and staging, defining the clear boundaries of tumor mass, monitoring disease progression and recurrence, assessing the effectiveness of therapies, and detecting drug resistance. A more immediate and achievable near-term goal is perhaps the seamlessly interfacing of microfluidic devices with conventional benchtop laboratory processes through modular “plug and play” platforms [75]. Moreover, the integration of novel materials (such as paper or thread) with low-cost fabrication technologies will provide some of the most promising developments in POC diagnostics in the coming decade [76]. These new approaches offer the promise of more rapid prototyping with less investment in capital equipment as well as greater flexibility in design.

Although the outlook of microfluidic technology is promising, the journey towards this lofty goal will be an extremely exciting and challenging one. There is a pressing need for a genuine collaborative and interdisciplinary effort, spanning from fundamental academic research to the commercial pathway towards translational biomedical technology.

## Abbreviations

LOC: Lab-on-a-Chip; μTAS: micro-total analysis systems; CAE: Capillary array electrophoresis; PCR: Polymerase chain reaction; IHC: Immunohistochemistry; MS: Mass spectrometry; ESI: Electrospray ionization; MALDI: Matrix-assisted laser desorption ionization; FACS: Fluorescence-activated cell sorting; MACS: Magnetically-activated cell sorting; DACS: Dielectrophoresis (DEP)-activated cell sorter; FADS: Fluorescence-activated droplet sorter; μFCM: Microfluidic flow cytometry; WGA: Whole genome amplification.

## Competing interests

Both authors declare that they have no competing interests.

## Authors’ contributions

Both LY and QW participated in the design of the manuscript and drafted the manuscript. Both authors read and approved the final manuscript.
